# Global reporting on TB in children and adolescents: how far have we come and what remains to be done?

**DOI:** 10.5588/ijtldopen.23.0529

**Published:** 2024-01-01

**Authors:** S. Verkuijl, M. Bastard, A. Brands, K. Viney, T. Masini, F. Mavhunga, K. Floyd, T. Kasaeva

**Affiliations:** Global Tuberculosis Programme, World Health Organization, Geneva, Switzerland

**Keywords:** global reporting, TB surveillance, children, adolescents, tuberculosis

**Figure 1.**
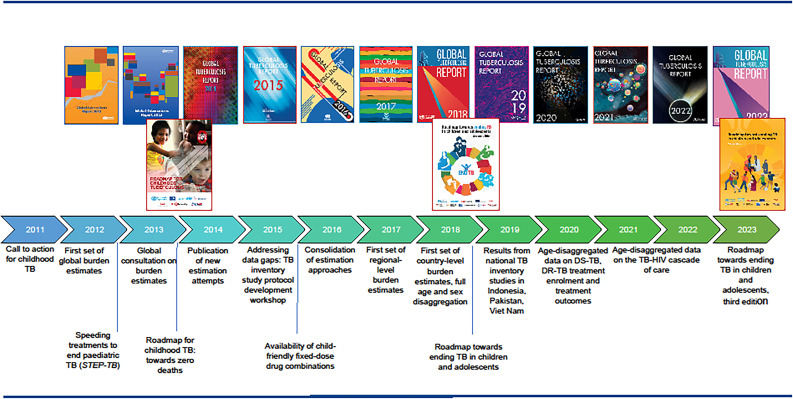
Global milestones in reporting on TB in children and adolescents, 2011–2022.

In September 2023, the second United Nations High-Level Meeting on the Fight Against Tuberculosis (UN HLM) provided an important opportunity to reflect on progress towards ending TB in children and adolescents, based on the most recent data reported to the WHO.^1,2^ Following a ‘Call to action for childhood TB’ in 2011,^3^ the availability of surveillance data and estimates of the burden of TB disease in children has improved and expanded (Figure 1). The Roadmap towards ending TB in children and adolescents,^4^ launched alongside the first UN HLM in 2018, provided an agenda for scaling up interventions for children (<10 years) and adolescents (10–19 years). It also highlighted the main gaps related to data collection, reporting and analysis. The third edition of the Roadmap towards ending TB in children and adolescents (published 14 November 2023), provides actions to further expand and improve data collection, reporting and use.^5^ Since 2020, the WHO has requested countries with case-based digital surveillance systems to report data on national TB notifications in five age groups (0–4, 5–9, 10–14 and 15–19 years), the number of children and young adolescents (0–14 years) started on treatment for multidrug- and rifampicin-resistant TB (MDR/RR-TB), and overall treatment outcomes for children and young adolescents.^1^ Updated WHO guidance on TB surveillance strongly encourages countries to transition to digital case-based surveillance systems, with a specific focus on enabling age-disaggregated reporting.^6^ In 2022, the WHO published consolidated guidelines on the management of TB in children and adolescents,^7^ along with an operational handbook,^8^ with practical implementation guidance on the full cascade of TB-related care. The guidance covers diagnostic approaches, shorter treatment of non-severe drug-susceptible TB and TB meningitis, use of bedaquiline and delamanid in children with MDR/RR-TB of all ages, and decentralised, family-centred and integrated models of TB care for the delivery of TB services. An estimated 1.25 million children and young adolescents (95% uncertainty interval [UI] 1.2–1.3 million) fell ill with TB in 2022; 47% of these were children aged ˂5 years.

**Figure 2.**
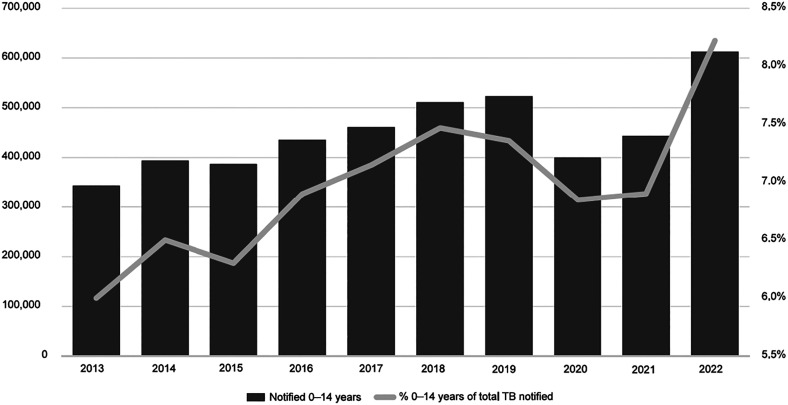
Trends in case detection in children and young adolescents (<15 years), 2013–2022.

This age group makes up 12% of the total TB incidence of 10.6 million.^1^ In 2022, an estimated 214,000 children and young adolescents (<15 years) died from TB (95% UI 190,000–239,000), which means that each day, almost 600 children and young adolescents lose their lives to this preventable disease. In 2022, 16% of the people who died from TB worldwide were children and young adolescents, a disproportionate percentage compared to the disease burden in this age group. Among all TB-related deaths in children and young adolescents, 31,000 (95% UI 26,000–37,000) (14%) were in those with HIV, 76% of HIV-negative deaths were in children aged ˂5 years.^1^ 96% of deaths occur in children and young adolescents who did not access TB treatment.^9^ The negative impact of the COVID-19 pandemic on overall global TB notifications has been well described.^10–12^ The decrease in notifications between 2019 and 2020 was highest in the youngest children (28% in children under 5, 21% for 5–14 year olds, compared to 18% in those aged ≥15).^13,14^ In 2021, recovery was also slowest in the youngest age group. A total of 613,000 children and young adolescents with TB were notified by national TB programmes in 2022; 8.2% of the total notifications of people with a new or relapse episode of TB.^1^ These data suggest that notifications in this age group are back on track, with the highest number ever reported (Figure 2). However, compared to estimated incidence, only 49% were diagnosed and reported. The proportion of people with TB who were not diagnosed or not reported in 2022 was highest in the youngest age groups: 58% for children under 5, compared to 45% in older children and young adolescents and 30% for ages ≥15.^1^ Since 2018, countries have reported data on treatment initiation for MDR/RR-TB in children, and between 3,200 and 5,600 children and young adolescents have been started on second-line TB treatment every year.^1^ Compared to the estimated 25,000–32,000 children and young adolescents with MDR/RR-TB,^15–17^ only 3,900 (12–16%) were diagnosed and treated in 2022. TB treatment outcomes in children and young adolescents are generally favourable; for the 2021 cohort (reported by 136 countries for 70% of notifications in that year), treatment success was 91%.^1^ Some sub-groups, such as children with HIV and those with malnutrition, may have less favourable outcomes.^18,19^ For drug-resistant TB, an individual TB participant database meta-analysis (including published and unpublished data up to 2020) reported a treatment success rate of 80% in over 7,000 children and young adolescents treated for MDR/RR-TB, with a mortality rate of 9% (Garcia-Prats, personal communication, 2023). In 2022, 20 out of 30 countries with the highest burden of TB-HIV reported data on the TB-HIV cascade of care for children and young adolescents. Of almost 390,000 children and young adolescents notified with TB, 260,000 (67%) had a known HIV status (newly or previously tested); 4.4% were HIV-positive. Out of almost 12,000 children and young adolescents with TB-HIV co-infection, just under 10,000 (86%) were on antiretroviral treatment.^1^

In 2022, 590,000 household contacts aged ˂5 years started TB preventive treatment (TPT). Although this represented a 40% increase compared with 2021, only 37% of the 1.56 million eligible child contacts aged under 5 received TPT. A total of 1.3 million household contacts aged ≥5 years started TPT in 2022, representing a four-fold increase compared with 2021. Data on the cascade of TB preventive care for all age groups show a big gap between the number of household contacts screened for TB disease and the number starting TPT. In 2022, 600,000 people started shorter rifamycin-containing TPT regimens in 74 countries; 58 countries were using the 3-month daily regimen of rifampicin and isoniazid; 56 countries reported using the 3-month weekly regimen of rifapentine and isoniazid (which can be used in ages ≥2, although no child-friendly formulation of rifapentine is available as yet).^1^

Five years after the first UN HLM on TB, progress against the targets for children and adolescents has been insufficient. Only 71% of the target to provide treatment to 3.5 million children and young adolescents has been achieved (compared to 84% of the 40 million target for all ages); 19% of the target to provide treatment for 115,000 children and young adolescents with MDR/RR-TB has been achieved (compared to 55% of the 1.5 million target for all ages). On the positive side, the target for provision of TPT to 6 million people living with HIV was exceeded by 5 million. This includes children and adolescents, although age-disaggregated data are not reported to WHO. Despite this achievement, progress against the targets for TPT in child contacts under 5 years (who are most at risk of progression from TB infection to disease) has been unsatisfactory, with only 2.2 million of the 4 million target (55%) achieved. Progress has been slowest in household contacts aged ≥5 years, with only 10% of the 20 million target achieved.^1^ In September 2023, at the second UN-HLM on TB, member states renewed their commitment to address TB in children and adolescents. The updated objectives comprise achieving a 90% coverage of the estimated number of people who develop TB, encompassing both diagnosis and treatment from 2023 to 2027. This effort will involve administering treatment to a potential 45 million individuals, including approximately 4.5 million children and 1.5 million individuals with MDR/RR-TB. The political declaration also aims to provide TPT to 90% of those at high risk of TB, which includes 30 million household contacts of TB patients, including children, and 15 million people living with HIV, totalling a target of 45 million individuals by 2027.^20^

Despite important progress in the diagnosis of TB, shorter and improved regimens for the prevention and treatment of drug-susceptible and drug-resistant TB, and improved service delivery models for children and adolescents, the available data show that much more needs to be done before we come close to eliminating TB in these age groups. Relevant WHO guidance should be widely implemented to improve equitable access to child-friendly screening, prevention, diagnosis and treatment services, and management of TB-related disability, as part of comprehensive integrated primary healthcare. Active approaches to contact investigation are critical to ensure linkage to diagnostic evaluation and prevention. Technical working groups play an important role in planning, implementation and monitoring of these interventions. They also need to ensure that all relevant stakeholders are engaged, including child health, nutrition and HIV programmes, civil society, affected communities, paediatric associations and implementing and funding partners. Alignment of age groups used in clinical trials with clinical age definitions (children <10 years, adolescents 10–19 years) is important for harmonised policy recommendations and implementation at the country level. Dedicated investments in TB research and development for children and adolescents, in line with their disease risk and burden, are needed to move towards the collective goal of ending TB in children and adolescents.
